# Subclavian artery cannulation provides better myocardial protection in conventional repair of acute type A aortic dissection: experience from a single medical centre in Taiwan

**DOI:** 10.5830/CVJA-2015-056

**Published:** 2016

**Authors:** Po-Shun Hsu, Chien-Sung Tsai, Yi-Ting Tsai, Chih-Yuan Lin, Chung-Yi Lee, Hong-Yan Ke, Yi-Chang Lin, Chien-Suang Tsai, Jia-Lin Chen

**Affiliations:** Division of Cardiovascular Surgery, Department of Surgery, Tri-Service General Hospital, National Defense Medical Centre, Taipei, Taiwan; Division of Cardiovascular Surgery, Department of Surgery, Tri-Service General Hospital, National Defense Medical Centre, Taipei, Taiwan; Division of Cardiovascular Surgery, Department of Surgery, Tri-Service General Hospital, National Defense Medical Centre, Taipei, Taiwan; Division of Cardiovascular Surgery, Department of Surgery, Tri-Service General Hospital, National Defense Medical Centre, Taipei, Taiwan; Division of Cardiovascular Surgery, Department of Surgery, Tri-Service General Hospital, National Defense Medical Centre, Taipei, Taiwan; Division of Cardiovascular Surgery, Department of Surgery, Tri-Service General Hospital, National Defense Medical Centre, Taipei, Taiwan; Division of Cardiovascular Surgery, Department of Surgery, Tri-Service General Hospital, National Defense Medical Centre, Taipei, Taiwan; Division of Cardiovascular Surgery, Department of Surgery, Taoyuan Armed Forces General Hospital, National Defense Medical Centre, Taoyuan, Taiwan; Department of Anesthesia, Tri-Service General Hospital, National Defense Medical Centre, Taipei, Taiwan

**Keywords:** acute aortic dissection, axillary artery cannulation, femoral artery cannulation, T-graft peripheral cannulation

## Abstract

**Background:**

Although many reports have detailed the advantages and disadvantages between femoral and subclavian arterial cannulations for acute aortic dissection type A (AADA), the confounding factors caused by disease severity and surgical procedures could not be completely eliminated. We compared femoral and subclavian artery cannulation and report the results for reconstruction of only the ascending aorta.

**Methods:**

From January 2003 to December 2010, 51 AADA cases involving reconstruction of only the ascending aorta were retrospectively reviewed and categorised on the basis of femoral (*n* = 26, 51%) or subclavian (*n* = 25, 49%) arterycannulation. Bentall’s procedures, arch reconstruction and hybrid operations with stent-grafts were all excluded to avoid confounding factors due to dissection severity. Surgical results, postoperative mortality, and short- and mid-term outcomes were compared between the groups.

**Results:**

Subclavian cannulation had a lower incidence of cerebral and myocardial injury and lower hospital mortality than femoral cannulation (8 vs 34%, *p* = 0.04). Ventilation duration as well as intensive care unit (ICU) and hospital stay were also shorter with subclavian cannulation. Risk factors for hospital mortality included pre-operative respiratory failure (odds ratio: 12.84), peri-operative cardiopulmonary bypass (CPB) time > 200 minutes (odds ratio: 13.49), postoperative acidosis (pH < 7.2, odds ratio: 88.63), and troponin I > 2.0 ng/ml (odds ratio: 20.08). The overall hospital mortality rate was 21%. The 40 survivors were followed up for three years with survival of 75% at one year and 70% at three years.

**Conclusions:**

Our results show that subclavian cannulation had a lower incidence of cerebral and myocardial injury as well as better postoperative recovery and lower hospital mortality rates for reconstruction of only the ascending aorta.

## Background

Surgical repair of acute aortic dissection type A (AADA) is always a significant challenge to cardiovascular surgeons. The key issues involved in this type of procedure include establishing adequate extracorporeal circulation, repairing the torn intima and friable aortic wall, and protecting vital organs, especially the brain, from ischaemia.

In the past two decades, there have been many debates regarding the use of femoral or axillary artery cannulation.^1^ Methods used may vary according to the extent of dissection, which may introduce a major statistical error when comparing cannulation sites. In this retrospective study, we excluded cases involving the arch and Bentall’s procedure and identified 51 patients in whom reconstruction of only the ascending aorta was performed. We analysed short- and mid-term results between the groups formed on the basis of femoral and subclavian cannulation. In addition, we predicted risk factors for mortality based on Kaplan–Meier survival curve results.

## Methods

Our study included 51 patients who were diagnosed with uncomplicated AADA, including DeBakey type I and II, via computerised tomography (CT) angiography, and had undergone simple reconstruction of the ascending aorta between 2003 and 2010. Bentall’s or David’s procedure was excluded if the intimal tear extended into the coronary ostium or aortic valve. Arch reconstruction was also excluded if any intimal tear was detected over the greater curve after aortotomy or if dissection of any one of the arch branches was confirmed on computerised tomography (CT) angiography.

In total, we excluded three cases of David’s procedure, seven cases of Bentall’s procedure, and nine cases of arch reconstruction, as well as two patients who underwent combined Bentall’s procedure and arch reconstruction. In other words, if no intimal tear was discovered over the aortic root or the arch, simple reconstruction of the ascending aorta was done and the patient was enrolled in our data set. In total there were 21 cases of DeBakey type I and 30 of DeBakey type II (Table 1). Of these 51 cases, seven patients underwent concomitant re-suspension of the aortic valve due to mild aortic regurgitation, noted during pre-operative echocardiography.

**Table 1:** Disease characteristics, clinical presentation and intra-operative variables

*Parameters*	*Total, n (%)*	*Femoral group, n (%)*	*Subclavian group, n (%)*	*p-value*
Total number	51	26 (100)	25 (100)	
DeBaykey type I	21 (41)	12 (46)	9 (36)	
DeBaykey type II	30 (59)	30 (59)	16 (64)	
Age (mean ± SD)	59.0 ± 14.0	60.9±13.7	57.0 ± 14.4	0.33
Gender (male)	38 (74)	20 (77)	18 (72)	0.687
Cerebral vascular accident	1 (2)	1 (4)	0 (0)	1.00
Coronary artery disease	8 (15)	4 (15)	4 (16)	1.00
Diabetes	7 (13)	4 (15)	3 (12)	1.00
Hypertension	42 (82)	23 (88)	19 (76)	0.24
PAOD	1 (2)	0 (0)	1 (4)	0.98
Congestive heart failure	13 (25)	8 (30)	5 (20)	0.37
COPD	6 (11)	2 (7)	4 (16)	0.62
Shock	12 (23)	7 (27)	5 (20)	0.56
Haemopericardium	14 (27)	10 (38)	4 (16)	0.138
Aortic regurgitation	7 (13)	6 (23)	1 (4)	0.11
Cerebral ischaemia	2 (4)	2 (7)	0 (0)	0.48
Respiratory failure	11 (21)	6 (23)	5 (20)	0.78
Visceral ischaemia	6 (12)	3 (11)	3 (12)	0.95
Renal ischaemia	11 (21)	6 (23)	5 (20)	0.78
Limb ischaemia	4 (8)	2 (7)	2 (8)	0.96
Operation time (h)	7.13 ± 1.60	7.53 ± 1.72	6.72 ± 1.39	0.07
Cardiopulmonary bypass time (min)	184.9 ± 57.1	177.9 ± 62.1	192.2 ± 51.6	0.37
Heart ischaemic time (min)	95.9 ± 33.6	97.0 ± 34.5	94.8 ± 33.3	0.81
Circulatory arrest time (min)	32.2 ± 5.4	33.8 ± 5.1	30.6 ± 5.3	0.03
Brain ischaemic time (min)	-	33.8 ± 5.1	0	0.00

PAOD, peripheral arterial occlusion disease, COPD, chronic obstructive pulmonary disease.

The patients were divided into two groups based on their arterial cannulation site. We collected the data retrospectively and focused on mortality rate and short- and mid-term results between the two cannulation groups. Operative mortality was defined as death within 30 days after surgery. Of the survivors, we compared ventilator-dependent days, intensive care unit (ICU) stay, and hospital stay between the two subgroups. The patients were scheduled for follow-up CT angiography every three months in the first year and then every year for the next two years.

Demographics and pre-operative characteristics of all 51 patients (38 men and 13 women) are summarised in Table 1. The mean age of the patients was 59.0 ± 14.0 years (median: 60.5; range: 33–87). All surgeries were performed on an emergency basis within 12 hours of the onset of symptoms. Forty-two patients (82%) had hypertension, 13 (25%) had congestive heart failure, and seven (13%) had diabetes. With regard to clinical presentations (Table 1), 12 patients (23%) were in shock (systolic blood pressure < 90 mmHg), 14 (27%) had haemopericardium, and 11 (21%) had respiratory failure with ventilator support. Two patients developed cerebral ischaemia (4%), six developed visceral ischaemia (12%), 11 developed renal ischaemia (21%), and four developed lower limb ischaemia (8%).

## Surgical technique

Before 2005, our cannulation strategy for repairing AADA tended towards more use of femoral artery cannulation. After 2005, the strategy switched to more use of subclavian artery cannulation because of developing familiarity with this technique. We routinely used 8- or 10-mm T-grafts for cannulation to avoid compromise of perfusion distally, and then performed median sternotomy and dual-stage venous cannulation over the right atrium.

Once cardiopulmonary bypass (CPB) was initiated, profound hypothermia was induced until the bladder or oesophageal temperature was less than 18°C. At the same time, protective adjuncts, such as barbiturates, steroids, ice packed around the head, and steep Trendelenburg positioning, were employed for cerebral protection. Aortic clamping was abandoned to avoid injury to the fragile intima. Once the temperature was below 18°C, hypothermic circulatory arrest without retrograde cerebral perfusion was used in the femoral cannulation group, while antegrade selective cerebral perfusion with arterial flow of 8–10 ml/kg/min was used in the subclavian cannulation subgroup.

Aortotomy approximately 3 cm above the sinus of Valsalva was performed, and the aortic root and arch were carefully inspected to determine the optimal procedure for each case. If arch reconstruction, Bentall’s procedure or David’s procedure was required, these patients were excluded from the study. St Thomas cardioplegic solution was directly infused via both coronary ostia for myocardial protection. Distal anastomosis with a Hemashield graft and sandwich procedure with Teflon felt strips for reinforcement of the diseased aorta was performed first, after which the perfusion was converted through the ascending graft and was initiated to minimise systemic ischaemic time. Proximal anastomosis was subsequently performed, followed by rewarming, adequate de-airing, and weaning of the extracorporeal circulation.

The intra-operative variables are summarised in Table 1. Overall, the mean CPB time was 184.9 ± 57.1 min, the mean heart ischaemic time was 95.9 ± 33.6 min, and the mean circulatory arrest time was 32.2 ± 5.4 min. In the femoral group, without selective antegrade cerebral perfusion, the mean brain ischaemic time was 33.8 ± 5.1 min.

## Statistical analysis

All statistical analyses were performed using SPSS software version 12 (SPSS, Chicago, IL, USA). Categorical variables are expressed as percentages and were evaluated with the χ^2^ test or Fisher’s exact test. Continuous variables are expressed as mean ± standard deviation and were evaluated using the Student’s *t*-test. Stepwise logistic regression analysis was used to determine the independent predictors of 30-day hospital mortality. Survival was calculated by the Kaplan–Meier method.

## Results

There were 11 (21%) deaths after surgery (Table 2), the causes of which included cardiac failure in six, visceral ischaemia in three, aortic re-dissection in one, and respiratory failure complicated by adult respiratory distress syndrome (ARDS) in one. Five patients (9%) had postoperative neurological complications, including transient neurological dysfunctions in three and permanent strokes in two. All five patients with neurological complications survived and were released from hospital. One stroke patient died of pneumonia with sepsis within three years. Twenty-six (51%) patients required transfusions of more than 500 ml packed red blood cells after surgery, and four (8%) underwent re-sternotomy for haemostasis. Furthermore, 12 (23%) had pneumonia, five (9%) had ARDS, and 22 (43%) developed acute renal failure. The subclavian group showed a significantly lower incidence of mediastinitis (*p* = 0.01), neurological dysfunction (*p* < 0.001), acute renal failure (*p* = 0.03), and mortality (*p* = 0.04) (Table 2).

**Table 2:** Post-operative general data and short-term outcomes

*Postoperative data*	*Total*	*Femoral group (n = 26)*	*Subclavian group (n = 25)*	*p-value*
pH		7.26 ± 0.15	7.35 ± 0.05	0.01
PaO_2_ (mmHg)		115 ± 92	136 ± 75	0.37
HCO_3_ - (mEq/dl)		20.5 ± 4.5	22.6 ± 3.1	0.06
Amylase (U/l)		316 ± 482	101 ± 159	0.04
Lipase (U/l)		98 ± 158	41 ± 57	0.09
GOT (U/l)		391 ± 1078	70 ± 113	0.14
GPT (U/l)		286 ± 890	33 ± 29	0.16
Troponin I (ng/ml)		7.5 ± 11.9	0.1 ± 0.3	0.003
CRP (mg/dl)		10.9 ± 8.0	5.4 ± 3.5	0.003
Albumin (g/dl)		2.9 ± 0.4	3.3 ± 0.4	0.001
*Short-term outcomes*				
Total number, n (%)	51	26 (100)	25 (100)	
Transfusion > 500 ml, n (%)	26 (51)	15 (57)	11 (44)	0.32
Resternotomy, n (%)	4 (8)	2 (7)	2 (8)	1.00
Mediastinitis, n (%)	2 (4)	2 (7)	0 (0)	0.01
Neurological dysfunction, n (%)	5 (9)	5 (19)	0 (0)	0.001
Pneumonia, n (%)	12 (23)	8 (27)	4 (16)	0.36
ARDS, n (%)	5 (9)	3 (11)	2 (8)	0.67
Acute renal failure, n (%)	22 (43)	15 (57)	7 (28)	0.03
Mortality, n (%)	11 (21)	9 (34)	2 (8)	0.04

The biochemical tests were recorded 24 hours after surgery. GOT, glutamate oxaloacetate transaminase; GPT, glutamate pyruvate transaminase; CRP, C-reactive protein; ARDS, acute respiratory distress syndrome.

In the 24-hour postoperative biochemistry data (Table 2), we found metabolic acidosis and hyperamylasaemia to be significantly higher (p < 0.05) in the femoral artery group. In addition, higher troponin I and C-reactive protein and lower albumin levels were also noted in the femoral artery group (p < 0.05). The survivors in the subclavian artery group had a shorter mean ventilator-dependent duration (6.0 ± 4.8 vs 6.4 ± 3.5 days) as well as ICU (8.9 ± 5.8 vs 13.3 ± 9.1 days) and hospital stay (18.8 ± 9.8 vs 34.1 ± 22.6 days) than those in the femoral artery group.

**Fig. 1.**
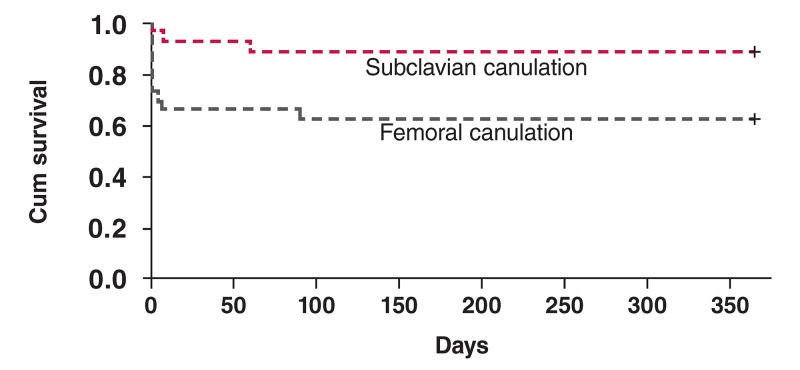
Kaplan–Meier survival curve in relation to the two groups.

The compared Kaplan–Meier survival curve for the two groups is shown in Fig. 1. All 40 survivors were followed up for three years with annual CT angiography. Two patients died within one year of surgery, one of sudden death and the other of a cerebral vascular accident. Two more patients died within the next two years, one of pneumonia with severe sepsis and the other following recurrent dissection of the aortic root.

**Fig. 2.**
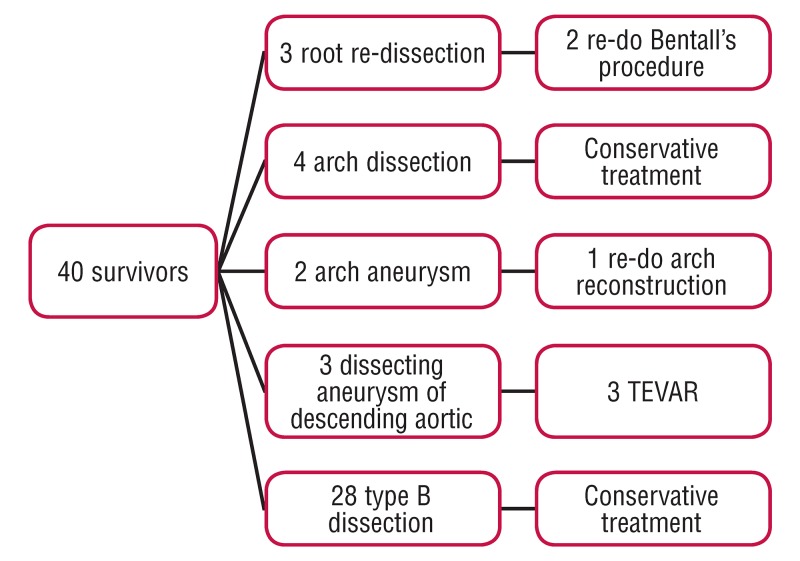
Outcomes of the annual CT angiography and follow-up intervention.

According to the outcomes of the annual CT angiography (Fig. 2), three root re-dissections were found; two underwent re-do Bentall’s operation and the other died of sudden death without re-operation. Four arch dissections without branch involvement were found and all four adopted conservative treatment. Two arch aneurysms were found and one underwent re-do arch reconstruction due to impending rupture. Three dissecting aneurysms of the descending aorta were found and all three underwent thoracic endovascular aortic repair (TEVAR). The other 28 were diagnosed with type B dissection and adopted conservative treatment. Overall survival was 75% at one year and 70% at three years.

Results of univariate and multivariate analysis are shown in Table 3. Logistic regression analysis revealed independent risk factors for hospital death as pre-operative respiratory failure, peri-operative CPB > 200 min, postoperative severe acidosis (pH 7lt; 7.2), and troponin I > 2.0 ng/ml.

**Table 3:** Significant risk factors associated with hospital mortality

	*Univariate analysis*		
*Variables*	*Survival group n (%)*	*Mortality group n (%)*	*p-value*
Total number	40 (100)	11 (100)	
Pre-operative			
Respiratory failure	2	6	< 0.001
Peri-operative			
Cardiopulmonary bypass time > 200 mins	10	9	< 0.001
Post-operative			
pH < 7.2	1	8	< 0.001
Troponin I > 2.0 ng/dl	4	8	< 0.001
Acute renal failure	11	11	< 0.001
	*Acute renal failure*		
*Variables*	*OR*	*95% CI*	*p-value*
Pre-operative			
Respiratory failure	12.84	1.48–111.0	0.020
Peri-operative			
CPB time > 200 min	13.49	1.29–140.1	0.029
Post-operative			
pH < 7.2	88.63	1.178–4.39	0.003
Troponin I > 2.0	20.08	1.37–293.4	0.013

AR, aortic regurgitation; CRP, C-reactive protein; CI, confidence interval; OR, odds ratio; CPB, cardiopulmonary bypass.

## Discussion

There is a trend towards cannulation of the axillary artery for extracorporeal circulation in patients with AADA,^1^-^4^ but the debate is ongoing and several possible reasons could explain this deficiency, including the following: (1) the urgency of AADA does not allow for complicated surgical techniques but instead requires a simple, rapid and safe approach to achieve rapid extracorporeal circulation; (2) the different individual situations demand an individual approach, and it is difficult to relate outcome to the cannulation site; (3) the number of procedures performed at each centre is rather small, especially in Asia, while a multicentre approach is not practicable owing to the different strategies practiced at different centres; and (4) most important of all, the severity of the AADA hinges on the location of the torn intima and the extent of the dissection, which also demands different types of procedures. In this study, we enrolled only cases involving ascending aorta reconstruction to avoid major statistical error that could have been introduced because of disease severity and procedure differences.

In this study, fewer postoperative complications and lower mortality rate were detected in the subclavian artery group (Table 2). Undoubtedly, the lower incidence of neurological dysfunction could be attributed to selective cerebral perfusion through subclavian cannulation.^5^ We also found that subclavian artery cannulation provided better perfusion for other visceral organs (Table 2).

Two possible hypotheses may elucidate why. First, according to the Hagen–Poiseuille law,^6^ the pressure drops in a fluid flowing through a long cylindrical pipe. So under constant blood flow, cardiac and sternal perfusion may not be adequate with femoral cannulation, especially when the aorta is dissected. Inevitably, sternitis and myocardial injury would be higher in the femoral cannulation group. Second, we assume that subclavian cannulation would pump major blood flow into the true lumen, while femoral cannulation pumps more blood into the false lumen.

Based on our evaluation, femoral cannulation introduced more retrograde dissection and exacerbated perfusion of the visceral organs. Survivors who underwent subclavian cannulation had better recovery during the postoperative hospital course, which might be attributed to better perfusion of the visceral organs. In other words, subclavian rather than femoral cannulation could achieve a lower incidence of visceral malperfusion during surgeries for AADA.

Our univariate analysis of hospital mortality revealed many risk factors, including pre-operative respiratory failure, perioperative CPB time > 200 min, postoperative acidosis, troponin I > 2.0 ng/dl, and acute renal failure (Table 3). Myocardial injury has been thought to be a risk factor during major aortic surgery, particularly when the thoracic aorta is involved.^7^-^8^ Although femoral cannulation was eliminated as a risk factor by multivariate analysis, it seemed to have a trend towards increased mortality because it resulted in a higher postoperative troponin I level, which leads to increased peri-operative cardiac injury, which reached statistical significance in the multivariate analysis. The significance could be confirmed if more patients are enrolled in the future.

This study has several limitations. First, the cohort was relatively small, but despite this, we still identified an advantage in subclavian cannulation, which suggests a significant benefit for simple reconstruction of the ascending aorta. Second, the data were collected from 2003 to 2010. Surgical techniques and general postoperative care may have improved in the latter part of the study, which could explain the unusually high mortality rate of femoral cannulation in the earlier phase. Third, the study was retrospective and not randomised. More prospective, randomised, controlled trials should be designed to support our hypothesis.

## Conclusion

Cardiac failure and visceral malperfusion are both fatal complications of AADA surgery.^9^,^10^ In Christian and co-workers’ study, antegrade perfusion to the true lumen appeared to be associated with superior long-term survival after hospital discharge.^11^ Based on our evaluation, we believe subclavian cannulation could provide better perfusion, not only for the brain but also for the myocardium and other visceral organs, leading to lower mortality rates and better recovery following AADA procedures.
